# An Optimized Metabarcoding Method for *Mimiviridae*

**DOI:** 10.3390/microorganisms8040506

**Published:** 2020-04-02

**Authors:** Florian Prodinger, Hisashi Endo, Yasuhiro Gotoh, Yanze Li, Daichi Morimoto, Kimiho Omae, Kento Tominaga, Romain Blanc-Mathieu, Yoshihito Takano, Tetsuya Hayashi, Keizo Nagasaki, Takashi Yoshida, Hiroyuki Ogata

**Affiliations:** 1 Institute for Chemical Research, Kyoto University, Gokasho, Uji 611-0011, Japan; 2 Department of Bacteriology, Faculty of Medical Sciences, Kyushu University, 3-1-1 Maidashi, Higashi-ku, Fukuoka 812-8582, Japan; 3 Graduate School of Agriculture, Kyoto University, Kitashirakawa-Oiwake, Sakyo-ku, Kyoto 606-8502, Japan; 4 Laboratoire de Physiologie Cellulaire & Végétale, CEA, Univ. Grenoble Alpes, CNRS, INRA, IRIG, Grenoble, France; 5 Faculty of Science and Technology, Kochi University, Nankoku, Kochi 783-8502, Japan

**Keywords:** ‘Megaviridae’, *Mimiviridae*, DNA polymerase, MEGAPRIMER, primer cocktail

## Abstract

*Mimiviridae* is a group of viruses with large genomes and virions. Ecological relevance of *Mimiviridae* in marine environments has been increasingly recognized through the discoveries of novel isolates and metagenomic studies. To facilitate ecological profiling of *Mimiviridae*, we previously proposed a meta-barcoding approach based on 82 degenerate primer pairs (i.e., MEGAPRIMER) targeting the DNA polymerase gene of *Mimiviridae*. The method detected a larger number of operational taxonomic units (OTUs) in environmental samples than previous methods. However, it required large quantities of DNA and was laborious due to the use of individual primer pairs. Here, we examined coastal seawater samples using varying PCR conditions and purification protocols to streamline the MEGAPRIMER method. Mixing primer pairs in “cocktails” reduced the required amount of environmental DNA by 90%, while reproducing the results obtained by the original protocol. We compared the results obtained by the meta-barcoding approach with quantifications using qPCR for selected OTUs. This revealed possible amplification biases among different OTUs, but the frequency profiles for individual OTUs across multiple samples were similar to those obtained by qPCR. We anticipate that the newly developed MEGAPRIMER protocols will be useful for ecological investigation of *Mimiviridae* in a larger set of environmental samples.

## 1. Introduction

*Mimiviridae* is a family of nucleocytoplasmic large DNA viruses (NCLDVs), or the proposed order “Megavirales” [1]. The first member of the *Mimiviridae* family is the amoeba-infecting giant mimivirus (*Acanthamoeba polyphaga mimivirus*) with a particle diameter of 750 nm and a large genome encoding over 1,000 genes [2,3]. While this first-discovered member of *Mimiviridae* infects amoeba, other members infect unicellular algae or heterotrophic protists other than amoeba [4,5,6]. The genome sizes of alga-infecting *Mimiviridae* are mostly from 370 kb to 560 kb, but larger genomes up to 668 kb have also been reported [5,7]. Their virion sizes range from 140 nm to 310 nm [4,7]. The genome size of heterotrophic protist-infecting *Mimiviridae* ranges from 600 kb to 1,500 kb, and their icosahedral heads measure from 300 nm to 750 nm, though the tupanvirus virion has a long tail (~0.5 µm) in addition to its capsid [2,8,9].

*Mimiviridae* are thought to influence the population dynamics of eukaryotic microorganisms in marine environments because they prey on these hosts. Previous studies identified that a *Mimiviridae* virus (*Aureococcus anophagefferens virus*, AaV) was associated with the brown tide caused by its host pelagophytes [10,11]. Other members of *Mimiviridae* have been identified in haptophytes, which are capable of forming blooms, such as *Prymnesium parvum* [12] and *Haptolina ericina* [13,14,15]. The cosmopolitan green algae *Tetraselmis* (class Chlorodendrophyceae) is infected by Tetraselmis virus [5]. Members of the *Mimiviridae* family also infect bacteria-feeding protists such as *Cafeteria roenbergensis* [9] and choanoflagellates [6]. In the ocean, the abundance of *Mimiviridae* is comparable to that of eukaryotes [16]. Moreover, *Mimiviridae* show a higher taxonomic richness than bacteria in the ocean [17], despite being less abundant than bacteria [16]. These numerical features and the wide host ranges of *Mimiviridae* suggest that they are one of the key players in marine microbial ecosystems. However, little is known about their community structures and dynamics in different environments [18].

Effective methods for the characterization of the community structures of *Mimiviridae* and related viruses have been the amplification and sequencing (i.e., “meta-barcoding”) of highly conserved genes such as the major capsid protein genes, DNA polymerase family B (*polB*) and DNA mismatch repair protein genes [10,18,19,20,21]. Recently, Li et al. proposed a novel meta-barcoding method (i.e., the MEGAPRIMER method) to investigate *Mimiviridae* diversity and revealed a hitherto unknown level of *Mimiviridae* richness in environmental samples [22,23]. Their approach used a set of 82 degenerate primers, which were designed to cover diverse *Mimiviridae polB* genes identified in the *Tara* Oceans metagenomic dataset [22,23]. Analysis of a coastal water sample with the MEGAPRIMER method resulted in the identification of 5,595 non-singleton operational taxonomy units (OTUs) at 97% nucleotide identity [23]. The same approach has also revealed the *Mimiviridae* community structures in samples from hot spring freshwater, brackish water of the mangroves, and the Sea of Japan [22]. Although the development of the MEGAPRIMER method helped to characterize *Mimiviridae* communities more effectively and deeply than other methods such as shotgun metagenomics or metatranscriptomics, the original MEGAPRIMER method was time consuming due to the requirement of 82 individual PCR amplifications per sample [23]. In the first study [23], the authors sequenced only the amplicons that were visible in gel electrophoresis. However, this approach was subjective in deciding whether an amplification was successful or not. Therefore, in the second study [22], the authors omitted the gel visualization step and used all primer pairs by compromising on the yield of high quality reads due to inclusion of non-specific amplification [22]. However, 82 PCR amplifications for a sample demand a large amount of template DNA and can induce risk of experimental errors (e.g., pipetting mistakes, sample swaps or contamination). Many experimental steps may also lead to unexpected biases if one needs to process several samples (e.g., two samples cannot be simultaneously amplified in the same thermal cycler).

In this study, we attempted to improve the MEGAPRIMER method by streamlining the protocol of amplicon preparation and purification by mixing primer pairs in “cocktails”. We tested our new protocols on four coastal seawater samples from different locations and time periods. Based on the obtained sequences, we also designed real-time quantitative PCR primers to quantify major OTUs in these samples and compared the quantification with the barcoding results. Finally, we investigated the diversity of eukaryotes in eukaryotic size fractions for the same samples by 18S metabarcoding.

## 2. Materials and Methods

### 2.1. Seawater Sampling, Storage, and DNA Extraction

In this study we analyzed four seawater samples, one of which had been previously analyzed [23]. The previously analyzed seawater sample (4 L) was collected from a 5-m depth at the entrance of Osaka Bay, Japan (34°19′28”N, 135°7′15”E) on 30 October 2015 [23]. The other three seawater samples (each 10 L) were collected from a 5-m depth at three locations in the Uranouchi Inlet, Kochi Prefecture, Japan (i.e., Uranouchi Station “J”: 33°25′43.2”N 133°22′49.5”E on 6 July 2017; Uranouchi Station “F”: 33°26′33.6”N 133°24′41.8”E on 21 June 2017; Uranouchi Station “M”: 33°25′60.0”N 133°24′38.3”E on 10 November 2017). The Osaka Bay sample was filtered through a 3.0 µm-pore polycarbonate membrane filter (diameter 142 mm, polycarbonate; Merck, Darmstadt, Germany). One liter of the filtrate was further filtered through a 0.22 µm-pore filtration unit (Durapore^®^ PVDF Membrane Filters, PVDF, Merck). The samples from the Uranouchi Inlet were sequentially filtered through 3.0 µm (diameter 142 mm) and 0.8 µm (diameter 142 mm) membranes (polycarbonate, Merck). One liter of the filtrate was then further filtered through a 0.22 µm filtration unit (Sterivex, polycarbonate, Merck). The filters were stored at −80 °C until DNA extraction. DNA extraction was carried out by the Proteinase-K method for the 0.22 µm filtration units [24] and the xanthogenate-sodium dodecyl sulfate method for the 0.8 µm and 3 µm filters [25].

### 2.2. PCR Conditions, Amplicon Purification Protocols, and Sequencing

Eighty-two previously designed degenerate primer pairs (MEGAPRIMER; Supplementary Table S1) [23] were used either individually or in mixtures (i.e., “cocktails”) of primers for PCR amplifications. Three mixing strategies were employed (i.e., “MP5”, “MP10”, and “MP20”). MP stands for “MEGAPRIMER”, while the number indicates how many primer pairs were mixed in each cocktail (i.e., 5, 10, or 20 primer pairs) (Tables S2–S5). Pre-mixing of the primers reduced the necessary number of PCR amplifications. For example, when 20 primer pairs are mixed in one cocktail (i.e., MP20), five PCR amplifications are necessary for one sample, instead of 82 PCR amplifications per sample. The combination of primers in individual cocktails was designed based on “prevalence”, which is defined by the number of *Tara* Oceans metagenomic samples in which individual primer pairs detect amplicons by in silico PCR. An appropriate number of primer pairs were mixed (i.e., 5, 10, and 20 primer pairs for MP5, MP10, and MP20, respectively) so that the primers in a cocktail have similar level of “prevalence”. For MP10, we also used a second version of mixing (MP10 version 2, Table S4), which was designed according to estimated annealing temperatures of individual primer pairs, which range from 45 °C to 52 °C. The cocktails of MP10 version 2 were mixed so that the average annealing temperature of every cocktail was approximately 47 °C. This strategy is not expected to improve the efficiency (i.e., reduction of unspecific amplicons) but it distributes unspecific amplifications more evenly across cocktails, which simplified quality control during our protocol development. In another PCR strategy named MP1, we used primer pairs individually in 82 PCR amplifications.

In total, we performed eight sequencing runs. The first seven runs were allocated to sequencing of amplicons from Osaka Bay sample with either MP1, MP5, MP10, or MP20 PCR amplification protocols (i.e., run number 1–7 in Table 1). These runs resulted in relatively deep sequencing compared with the eighth run. The eighth sequencing run was assigned for other studies in addition to this study, and thus resulted in shallower sequencing for the individual samples. To distinguish realized sequencing depth, the dataset names for deep sequencing (i.e., the first to seventh runs) received the prefix “D” (deep sequencing), while the dataset names for shallow depth sequencing (i.e., the eighth run) received the prefix “S” (shallow sequencing). The prefix is followed by the sampling location (“OB” for Osaka Bay, and “UF”, “UM”, and “UJ” for Uranouchi F, M and J stations, respectively), the employed cocktail method (either MP1, MP5, MP10, or MP20) and finally the replicate number (1 or 2). D-OB-MP1-2 is given replicate number “2”, despite that there is no replicate number “1”, because this sample was prepared at the same time as other replicate “2” of D-OB- samples (i.e., D-OB-MP5/10/20-2).

We used different PCR conditions and purification protocols, which are detailed in Supplementary Table S6 (denoted protocol number 1–5 in Table 1). Major differences in these experimental conditions are as follows: the PCR conditions for deep sequencing runs of MP5, MP10, and MP20 (sequencing run numbers 1–3 and 5–7 in Table 1) were 0.625 ng of sample DNA (0.025 ng µL^−1^ final concentration), 8 µmol L^−1^ of the respective primer cocktail, and 50% KAPA Hifi Hotstart ReadyMix (Roche, Basel, Switzerland). Amplicon cleanup was performed using Agencourt AMPure XP beads (Beckman Coulter, Inc., Brea, CA) following Illumina’s library preparation protocol [26]. MP1 run of this study (run number 4) was conducted using the same protocol as the cocktail methods (MP5, MP10, and MP20), but with 1 ng of template DNA per PCR (0.04 ng µL^−1^). The thermal cycler (Thermal Cycler Dice Real Time System, Takara Bio Inc., Shiga, Japan) was programmed to start with 95 °C (3 min), followed by 32 cycles of 94 °C for melting (30 s), 54 °C for annealing (30 s), and 72 °C for elongation (30 s). The final elongation step was 4 min at 72 °C. The amplicons for shallow depth sequencing (run number 8 in Table 1) were prepared with the MP10 cocktails (either version 1 or 2) with the exception of one sample (S-UJ-MP1) amplified by MP1. The PCR cycle was the same for the shallow depth runs as for the deeply sequenced samples, but the concentrations of primers and templates as well as clean up protocols varied (Table S6). Amplicon cleanup was recognized as an important step, because for some samples we observed unspecific short amplicons, which reduced the yield of target sequences (with an expected size range from 374 bp to 590 bp including adapters and primers [23]). We thus used two clean up protocols for the samples that were subjected to the shallow depth sequencing run as follows: ethanol precipitation with subsequent agarose gel extraction (2% agarose gel in TAE buffer, Wizard SV, Promega, Madison, WI) was used on five amplicon PCR products (protocol number 3 in Table 1); three amplicon PCR products (protocol numbers 1, 4, and 5 in Table 1) were purified with magnetic beads (Agencourt AMPure XP beads, Beckman Coulter, Inc.) according to the recommended clean up protocol (Table 1).

After amplicon purification, dual indices were attached according to Illumina’s library preparation protocol and subjected to paired-end sequencing (2 × 300 nucleotides) on the MiSeq platform. The purified amplicons were mixed in equal volumes to prepare the final library. The sequencing conditions were 10 pmol L^−1^ library with an internal standard of 50% phiX for the first seven sequencing runs and 10 pmol L^−1^ library with an internal standard of 25% phiX for the eighth run.

### 2.3. Computational Quality Control of Reads

Raw reads were processed with *Mimiviridae* Amplicon Processing System (MAPS) [23]. MAPS is composed of seven steps. First, Trimmomatic v 0.35 [27] was used to remove all reads with quality under 5 and a length under 40 nucleotides. In the second step, Cutadapt v 1.14 [28] was used to remove the degenerate primers with a default value of 10% error rate. Thirdly, FLASh v 1.2.11 [29] was used to merge paired-end reads with 10% error rate and a minimum overlap of 100 nucleotides. FLASh was used three consecutive times: the first time with the “innie” option, and the second time with the “outie” option. For the third time, an inhouse-sliding-window script, which finds the best settings for Trimmomatic on seemingly inutile reads, was used. FLASh was then used on the recovered reads with the “innie” option. Chimeric sequences were removed with UCHIME of QIIME (1.9.1) [30]. In the fourth step, cd-hit-est v 4.6.8 [31] was used with 100% identity to group all duplicate reads. The fifth step translated the nucleotide sequences into amino acid sequences. All reading frames were considered in this process and sequences containing stop codons were discarded. In the sixth step, the translated sequences were searched against a custom database of 10,406 microbial PolB and 1,007 giant virus PolB sequences using blastp v 2.5.0 (E-value < 10^−5^). All sequences with a best hit to non-viral sequences were discarded. In the last step, Mafft v 7.310 [32], Pplacer v1.1.alpha.19 [33], blastp and an inhouse python v 3.7.5 script were used to further filter out ambiguous sequences. The obtained sequences were placed in a reference tree of PolB sequences using Pplacer. Sequences that were not placed near *Mimiviridae* PolB reference sequences in the phylogenetic tree were discarded. Finally, a python script was used to trim sequences to retain only shared regions.

The resulting nucleotide sequences (the output of MAPS) were then merged into a single file and cd-hit-est (97% identity, v 4.6.8) [31] was used to form OTUs. Singleton OTUs were discarded from analysis. Plots were generated with either base R (v 3.4.2) [34], ggplot2 [35] or iNEXT [36]. In this study, we also included a previously generated dataset as D-OB-MP1-0 [23]. *Mimiviridae* PolB sequences were aligned with reference sequences using Mafft [32] and a phylogenetic tree was generated by FastTree [37]. Mafft and FastTree were used with default settings. Anvi′o was used for tree visualization [38]. The Jaccard dissimilarity and other diversity metrics were calculated by firstly subsampling with the *rrarefy* function of R’s vegan package (2.5-6) [39] to normalize the sequencing depth at the lowest read count (S-OB-MP10-1) and secondly by applying *vegdist*. The default *cmdscale* and *hclust* were used on the Jaccard dissimilarity to perform non–metric multidimensional scaling (NMDS) and hierarchical clustering, respectively.

### 2.4. qPCR Primer Design and Experiments

We selected the 10 most abundant OTUs from each of the 15 datasets to design qPCR primers, resulting in 58 OTUs after removing the overlap. The most abundant genotype of each of the OTUs was selected as the initial target. If the most abundant genotype represented less than half of the sequences in the corresponding OTU, the genotype was discarded from the list of the candidate targets. Consequently, we removed 15 OTUs from the candidate list. The most abundant genotypes of the 43 remaining OTUs were used to design specific primers using Primer3 [40,41], with an optimal primer size of 20 bp (minimum size, 18 bp; maximum size, 22 bp), and with a product size ranging from 50–250 bp. We discarded 20 OTUs that did not yield primers with the requirements above. The remaining 23 genotypes returned two to four qPCR primer pairs. We confirmed the specificity of the primers in silico using blastn search of all selected primer sequences against RefSeq, which resulted in the detection of no significant hits (E-value < 10^−4^). Additionally, all primer sequences were searched in the generated sequencing data for identical sequences. The primer set was discarded if primer pairs were suspected to be nonspecific (e.g., amplifying genotypes of OTUs that were not the target). After this screening, eight primer pairs targeting eight different OTUs remained as candidates for qPCR experiments (Table S7). We further checked specificity by recording dissociation curves after amplification. Two of the eight primer pairs showed multiple peaks in their dissociation curves and were not used for further analysis.

For the qPCR experiment, 6.25 µL of TB Green Premix Ex Taq™ II (Takara Bio Inc.), 1 µL of 20 µM reverse and forward primer mixture (final concentration 1.6 µM), and 1 µL of 1 ng µL^−1^ sample DNA (final concentration 0.08 ng µL^−1^) were diluted to 12.5 µL and amplified with 50 cycles of 20 s at 95 °C, 20 s at 55 °C, and 20 s at 72 °C, with fluorescence recorded during the last step. The limit of quantification (LoQ) was determined by the coefficient of variation (CV = 100.SD.mean^−1^) [42]. The CV was calculated from at least three measurements for every dilution of the standard serial dilution (10–10^7^ molecules). The LoQ was specified as the concentration of standards where the CV is at least 50% of the measured molecules or when 10 or less copies of DNA were detected. Target concentration was considered to be below the limit of detection (LoD) if the averaged measurement was less than one copy of DNA.

### 2.5. Metabarcoding Analysis of Eukaryotes

Eukaryotic diversity of the 3 µm fraction of the four seawater samples was analyzed by amplifying and sequencing the V8/V9 region of the 18S ribosomal RNA gene (18S rDNA). PCR was performed by mixing 12.5 µL 2× KAPA HiFi HotStart ReadyMix, 5 µL of 1 µmol L^−1^ forward primer (MiSeq adapter + “V8 F”: TCGTCGGCAGCGTCAGATGTGTATAAGAGACAG – ATAACAGGTCTGTGATGCCCT) [43], 5 µL of µmol L^−1^ reverse primer (MiSeq adapter + “1510”: GTCTCGTGGGCTCGGAGATGTGTATAAGAGACAG - CCTTCYGCAGGTTCACCTAC) [43], and 2.5 µL of template DNA (0.25 ng µL^−1^). The thermal cycler was programmed to hold 98°C for 3 min, and then proceed to 25 cycles of 98°C for 20 sec, 65°C for 15 sec, 72°C for 15 sec, and a final elongation step of 72°C for 10 min. Purification and clean-up of the PCR products were performed with Agencourt AMPure XP beads. Amplification success was confirmed by agarose gel electrophoresis. The dual indices were attached to the amplicons and the indexed amplicons were diluted to 2 nmol L^−1^, pooled, and loaded onto an Illumina MiSeq (Illumina, Inc., San Diego, CA) at a final concentration of 10 pM with 25% phiX spike-in. Paired-end sequencing (2 × 300 nucleotides) was conducted together with the MEGAPRIMER amplicons and other unrelated 18S and 16S amplicons in the eighth sequencing run.

We used QIIME 2 (version 2018.11.10) [44,45] to analyze the 18S amplicon sequence data with default settings unless specifically noted below. The reads were imported using a manually created input file (called “manifest file”) and primers were removed using cutadapt [28]. Vsearch was used for merging (minimum overlap, 35 bp; allowed mismatches in the forward and reverse read, 5) [46]. The merged reads were quality filtered, (minimum PHRED score of 10) and Vsearch was used again for dereplication and chimera checking against the SILVA 132 database [47]. OTUs were formed by clustering without reference at 99% identity. All singleton OTUs were discarded at this stage. The OTUs were then searched against the SILVA 132 ribosomal RNA database using QIIME 2′s feature-classifier [44] with 99% identity for taxonomic annotation. We kept only those OTUs that were taxonomically assigned at the phylum or lower levels and discarded those remaining. The OTU table was then exported and used to generate plots with R. Subsampling to 30,000 reads was performed with the *rrarefy* function of R’s vegan package to reduce the number of total reads for alpha diversity analysis. A list of major eukaryotic lineages was defined based on the taxonomic classification provided by SILVA (mostly level 4 and 5) and used to summarize the community compositions of eukaryotes (Supplementary Table S8).

### 2.6. Data and software availability

The raw reads generated in this study were deposited to DDBJ (Megaprimer amplicon data: DRA009129; 18S rRNA gene amplicon data: DRA009128). Processed sequence data as well as a recommended protocol for the MEGAPRIMER method are available from our ftp site (ftp://ftp.genome.jp/pub/db/community/MEGAPRIMER_papers). The MAPS pipeline is available on the FTP site: ftp://ftp.genome.jp/pub/tools/MEGAPRIMER.

## 3. Results

### 3.1. Mimiviridae Community Profiles were Coherent across Different Primer Cocktails

We generated 15 datasets of *Mimiviridae polB* amplicon sequences from four samples collected at four distinct locations (Osaka Bay and Uranouchi Inlet station F, J, and M) through either deep (run 1–7) or shallow (run 8) sequencing (Table 1). In total, 37 million raw paired-end reads were generated. Of these reads, 5.7 million reads were classified as of *Mimiviridae* origin (i.e., “MAPS-validated” *Mimiviridae polB* sequences; Table 2). The proportion of the MAPS-validated sequences to the total number of raw reads varied between 8% and 57% (26% on average). These *polB* sequences were grouped into 6,045 non-singleton OTUs at 97% sequence identity (11,591 OTUs if singletons are counted). The length of these *polB* sequences ranged from 104 bp to 523 bp with an average of 331 bp (SD: 27.4 bp).

**Figure 1:**
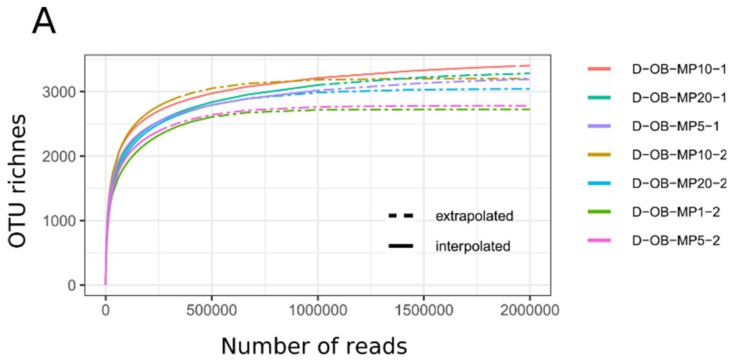

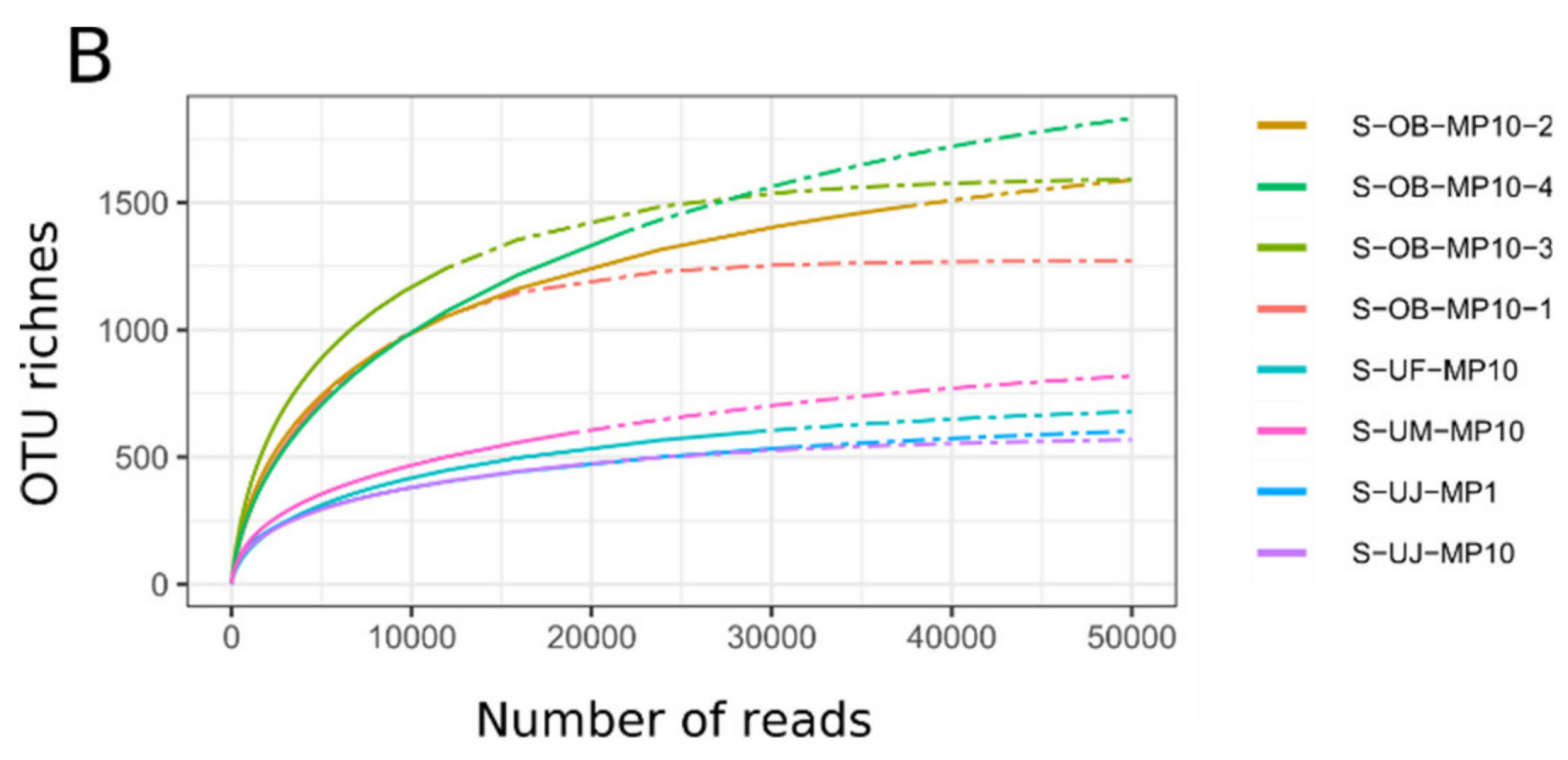
Rarefaction curves for the *Mimiviridae polB* operational taxonomic units (OTUs) from four samples. (**A**) Datasets produced by the deep sequencing runs. (**B**) Datasets produced by the shallow sequencing run. The rarefaction curves visualize the OTU and read counts detailed in Table 2.

In the deep sequencing experiment, each of the seven libraries generated 2.2×10^6^–10.7×10^6^ raw paired-end reads. From these sequences, we identified 273,153–1,916,193 *Mimiviridae polB* sequences (Table 2). After OTU clustering, each library comprised 2,426–3,396 non-singleton *Mimiviridae* OTUs (Figure 1A). In the shallow sequencing experiment, each of the eight libraries produced 34,860–96,149 raw paired-end reads, from which we identified 5,258–37,638 MAPS-validated *Mimiviridae polB* sequences. These *polB* sequences were classified into 470–1,487 non-singleton OTUs (Figure 1B). Therefore, the deep sequencing runs produced a larger number of OTUs compared with the shallow depth sequencing run for individual samples. This was also true for the datasets derived from the same Osaka Bay sample (2,426–3,396 OTUs for D-OB samples; 744–1,487 OTUs for S-OB samples). Indeed, as expected, the shallow depth sequencing failed to detect many of the OTUs identified in the deep sequencing runs.

**Figure 2:**
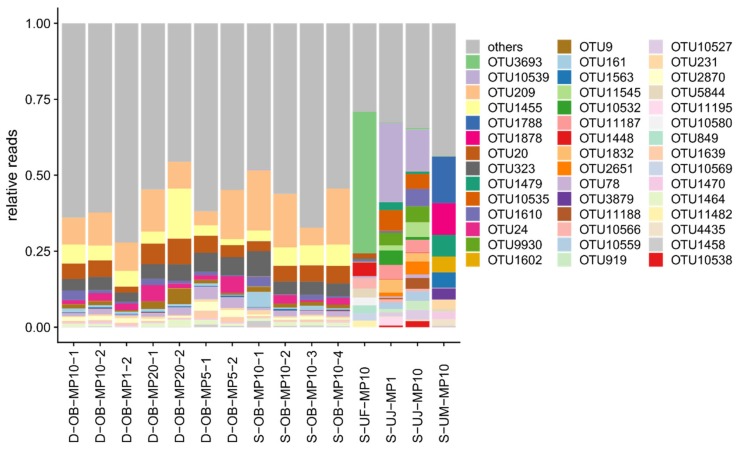
Relative frequencies of *Mimiviridae* OTUs across samples. Forty-four OTUs are represented by bars with colors if their relative frequencies reach at least 2% in any dataset, otherwise OTUs are grouped together in the “others” category.

**Figure 3:**
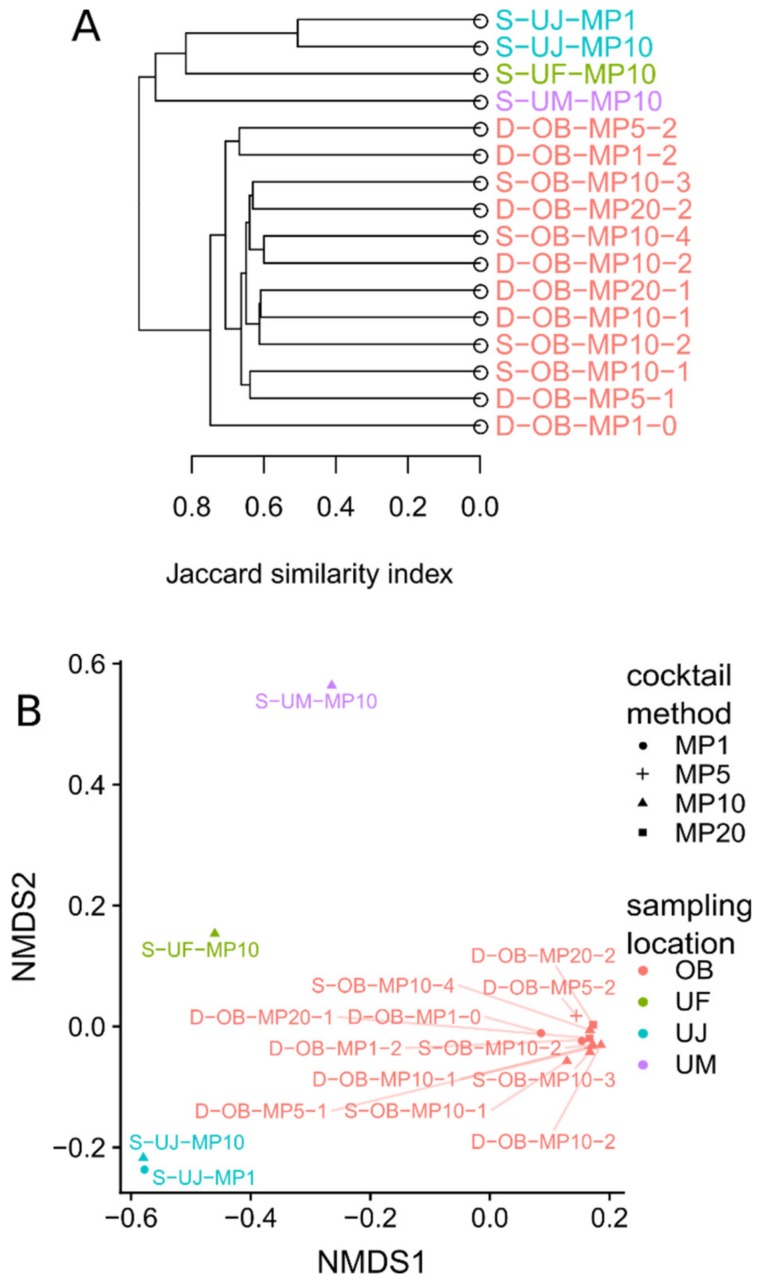
Jaccard Dissimilarity among replicated experiments and different samples. (**A**) Hierarchical clustering analysis of all *Mimiviridae* libraries. (**B**) Non–metric multidimensional scaling (NMDS) ordination of all MEGAPRIMER sequencing runs.

Although the total number of detected OTUs was dependent on the sequencing depth, the datasets for the single Osaka Bay sample showed comparable OTU profiles regardless of the primer cocktail strategy (i.e., MP1, MP5, MP10, MP20) or other differences in sample preparation protocols (Figure 2, Figure 3). Furthermore, the number of OTUs was comparable between sequencing depth-normalized datasets from the same sample (D/S-OB samples or S-UJ samples) (Figure S1). D-OB-MP1-0 was previously generated with the use of a subset of 82 MEGAPRIMER primer pairs (i.e., 58 selected primers). When normalized for sequencing depth, this dataset showed a similar number of OTUs with the 11 Osaka Bay datasets obtained in this study. D-OB-MP1-0 was also grouped with the other Osaka Bay datasets (Figure 3), although its OTU composition was slightly different from others (Figure 3B). The similarity between OTU profiles for the Osaka Bay samples was particularly pronounced for the profiles of abundant OTUs (Figure 2, Figure S2). Indeed, OTUs shared among the 11 Osaka Bay samples were found to be abundant. Specifically, 7% (397 OTUs) of all 5,657 OTUs from the Osaka Bay sample were shared among all 11 Osaka Bay datasets. Notably, this small number of OTUs represented a majority (82%) of all *Mimiviridae polB* sequences from the Osaka Bay datasets (Figure S3). In a similar manner, two Uranouchi datasets from one sample (S-UJ-MP1 and S-UJ-MP10) showed a relatively small dissimilarity value (0.37) and were placed closely in the NMDS plot based on the Jaccard dissimilarity (Figure 3). In contrast to these comparable OTU profiles for the datasets derived from same samples, the OTU profiles significantly differed across different samples (ANOSIM statistic = 1, *p* = 1×10^−5^; Figure 2, Figure 3).

**Figure 4:**
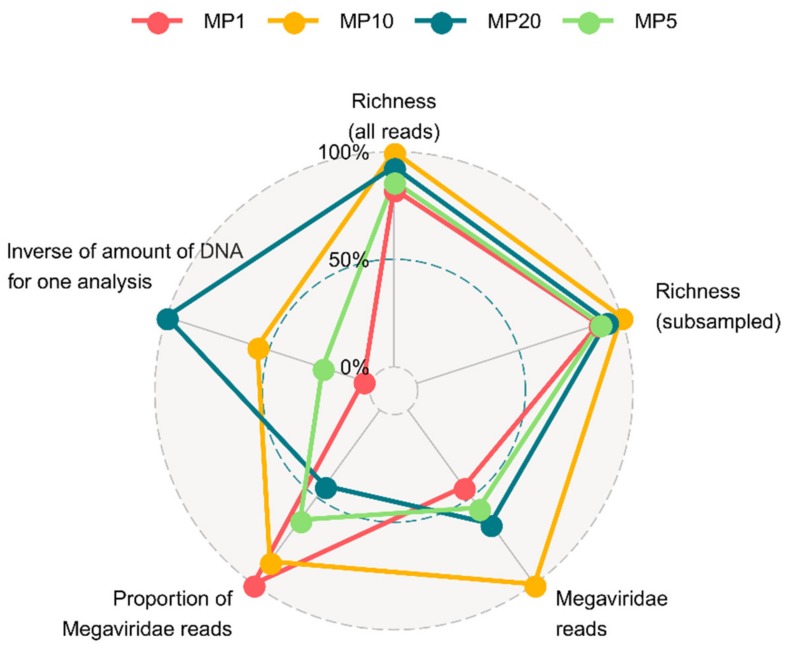
Radar chart assessing the effectiveness of different primer cocktail methods. The assessment is based on the deep sequencing run for the Osaka Bay sample (i.e., runs 1–7). Five axes represent i) total richness; ii) subsampled richness; iii) number of *Mimiviridae* reads; iv) proportion of the number of *Mimiviridae* reads among total number of raw reads (i.e., “usability”); v) inverse of the total amount of template DNA needed for one analysis.

We compared four strategies of primer cocktails (i.e., MP1, MP5, MP10, MP20) based on the results for the Osaka Bay sample (Figure 4). The OTU richness values obtained with these strategies were similar to each other, although MP10 generated a slightly larger number of OTUs than the other primer cocktail methods. However, individual strategies showed larger differences in terms of the numbers and proportions of MAPS-validated *Mimiviridae* reads. Obviously, they also differed in terms of the quantity of DNA required per analysis, arising from the differences in the number of amplicon preparation steps.

As for MP10, we used two versions of primer mixture (version 1 and 2) with additional variations in amplification protocols for the Osaka Bay sample with shallow depth sequencing (S-OB-MP10). The *Mimiviridae* community structures were similar with each other for these samples (Figure 3), although the combination of MP10.v2 and protocol 3 yielded only 5,258 *Mimiviridae* reads (S-OB-MP10-1) for unidentified reason. When we tried to amplify *Mimiviridae polB* sequences in the UJ sample with MP10 version 1 (with protocol 1 in Supplementary Table S6), purified amplicons were repeatedly found to be unsuitable for sequencing, because short unspecific amplification products remained after purification by magnetic beads. We thus employed gel extraction (with MP10.v2) and could reduce unspecific short amplicons for this sample. The same protocol and primers also worked for the other Uranouchi Inlet samples.

Finally, we found that *Mimiviridae* OTU richness was higher for Osaka Bay datasets compared with Uranouchi Inlet datasets after sequencing depth normalization (Figure 1, Figure S1). *Mimiviridae* richness after subsampling was 788 OTUs on average (ranging from 651 OTUs to 907 OTUs) for the Osaka Bay datasets, while the richness in Uranouchi Inlet samples was 334 OTUs on average (UF, 330 OTUs; UJ, 303 OTUs; UM, 370 OTUs).

### 3.2. Quantitative Assessment of Mimiviridae Operational Taxonomic Units (OTUs) and its Comparison with Meta-Barcoding Profiles

We quantified six abundant *Mimiviridae* OTUs in the four tested samples with the use of qPCR and compared the results with OTU profiles obtained through our MEGAPRIMER barcoding approach. The selected OTUs for this quantitative assessment were OTU1610, OTU231, OTU5844, OTU1788, OTU323, and OTU1458 (see Materials and Methods).

**Figure 5:**
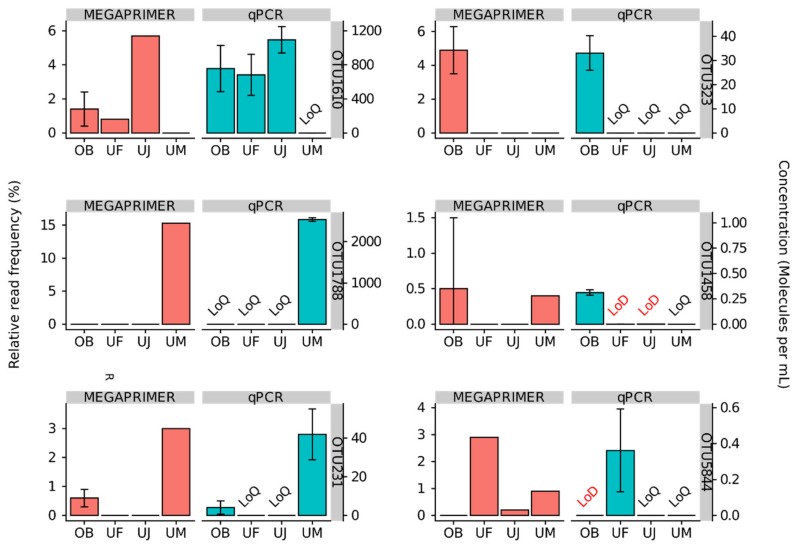
Relative read frequencies and qPCR quantification of selected six OTUs in four samples. The relative read frequency in the Osaka Bay sample was calculated by averaging all Osaka Bay sequencing results of this study. For Uranouchi Inlet samples, the relative read frequency in the S-UF-MP10 (UF), S-UJ-MP10 (UJ) and S-UM-MP10 (UM) datasets are shown. The error bar indicates one standard deviation. LoD, limit of detection; LoQ, limit of quantification.

Meta-barcoding by MEGAPRIMER may not be quantitative because individual *polBs* can be amplified by varying numbers of primer pairs [23]. In agreement with this expectation, relative frequencies assessed by the MEGAPRIMER barcoding were incongruent with the qPCR quantification for some OTUs (Figure 5). For example, the concentration of OTU1610 in the Osaka Bay sample was 756 ± 271 molecules mL^–1^, while this OTU showed an average relative frequency of 1.4 ±1 % in the barcoding datasets. In contrast, the concentration of OTU323 in the same sample was 33 ± 7 molecules mL^–1^, but this OTU showed an average relative frequency of 4.9 ± 1.4%, which is higher than the relative frequency of OTU1610.

Despite this limitation in the quantitativity of the MEGAPRIMER approach, the qPCR and MEGAPRIMER profiles were still similar to each other for individual OTUs across samples (Figure 5). OTUs that showed a concentration above 1,000 mL^−1^ in the qPCR assessment showed their relative abundance from 5.7% to 15.3% (10.5% on average; *n* = 2). Other OTUs that showed a quantifiable concentration (0.18 mL^−1^–756 mL^−1^) demonstrated relative abundance between 0.49% and 4.9% (2.0% on average; *n* = 7) in the sequence dataset derived from the same sample. OTUs that could not be quantified (i.e., below LoQ) with qPCR had a relative read abundance between 0% and 0.97% (0.13% on average; *n* = 12). OTUs that were not detected (i.e., below LoD) with qPCR had a relative read abundance between 0% and 0.0075% (0.0025% on average; *n* = 3) (Figure 5). OTU1610 and OTU231 were quantified in more than one sample by qPCR (OTU1610 in three samples; OTU231 in two samples). For these OTUs, the abundance ranks across samples were congruent between qPCR and meta-barcoding. Finally, albeit approximative, a statistically significant positive correlation was found between the relative read frequencies and qPCR molecular concentrations (Pearson’s r = 0.85, *p* = 3.7 × 10^–4^, excluding the OTUs below LoD; Figure S4).

### 3.3. Eukaryotic Communities

**Figure 6:**
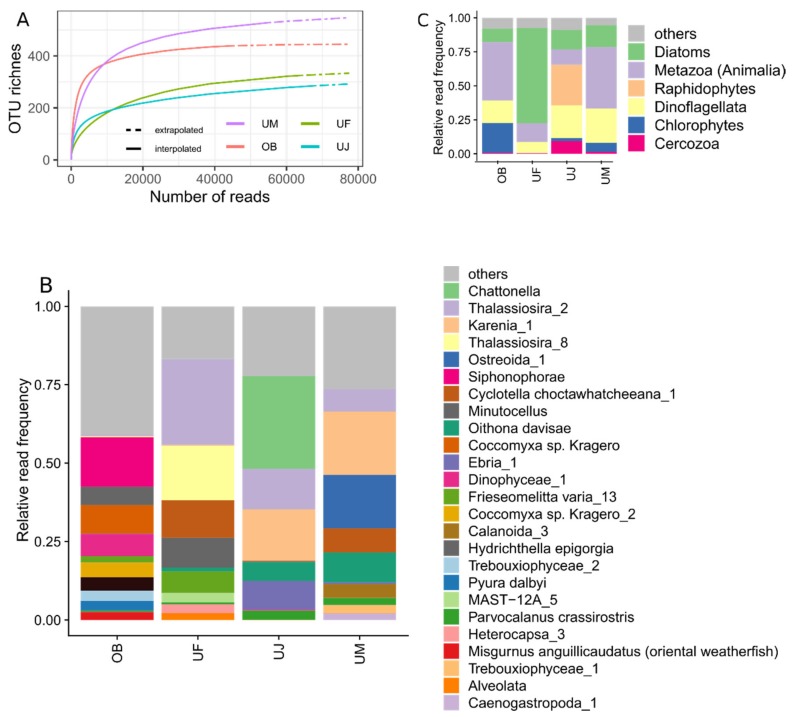
Eukaryotic communities in four samples. (**A**) Rarefaction curves for the number of OTUs in four samples based on 18S rRNA gene amplicon analyses. (**B**) Relative frequencies of eukaryotic OTUs across samples. OTUs are represented by bars with colors if their relative frequencies reach at least 2% in any dataset, otherwise OTUs are grouped together in the “others” category. (**C**) Relative frequencies of major eukaryotic lineages in four samples.

The *Mimiviridae* community of the Osaka Bay sample was on average twice as rich as that of the Uranouchi Inlet samples (Figure 1, Figure S1). To see if this difference was due to the differences in the eukaryotic community structures at the sampling sites, we performed 18S rRNA gene meta-barcoding for these samples (OB, UF, UJ, and UM samples). We generated 323,898 raw paired-end reads. After quality control, these raw reads resulted in 230,884 merged and taxonomically annotated reads, which were grouped into 1,156 non-singleton OTUs at a 99% sequence identity threshold (Table 3). The OB, UF, UJ, and UM samples yielded 439, 325, 285, and 528 OTUs, respectively (Table 3; Figure 6A). Depth-normalized Shannon’s diversity index was 4.1, 2.6, 2.8, and 3.3 for OB, UF, UJ, and UM, respectively.

OTU profiles were largely different between the four samples (Figure 6B). Dominant OTUs in the OB sample were annotated as Siphonophorae (the phylum Cnidaria,15.8%), the green algae *Coccomyxa* sp. (9.1%), Dinophyceae (7.1%), and the centric diatom *Minutocellus* (5.8%). The UF sample was dominated by centric diatoms (two OTUs for *Thalassiosira*, 44.7%; 1 OTU for *Cyclotella choctawhatcheeana*, 12.0%; 1 OTU for *Minutocellus*, 9.5%). The remaining Uranouchi Inlet samples (UJ and UM) contained abundant OTUs corresponding to toxic bloom-forming algae (the raphidophyte *Chattonella*, 29.6% in UJ; the dinoflagellate *Karenia*, 16.3% in UJ and 20.2% in UM). Centric diatoms were also observed (*Thalassiosira*, 12.9% in UJ, 7.2% in UM; *Cyclotella choctawhatcheeana*, 7.7% in UM).

These eukaryotic OTUs from the four samples (1156 OTUs) were classified into 72 major eukaryotic lineages (see Materials and Methods). Of these lineages, six lineages represented 91%–95% of the eukaryotic communities of the four samples (Figure 6C). These lineages were diatoms, metazoans, dinoflagellates, raphidophytes, chlorophytes, and Cercozoa (Rhizaria). Of these six lineages, only chlorophytes contained known hosts of isolated *Mimiviridae* [5,15], and they were found to show the largest relative frequency in the Osaka Bay sample. When the sequence-depth normalized richness for these six eukaryotic lineages were compared between the four samples, we did not find any clear clues about the high richness of *Mimiviridae* in the Osaka Bay sample (Figure S5). We also examined other lineages that contain known hosts of *Mimiviridae*, such as haptophytes, choanoflagellates, and Bicosoecophyceae [6,9,48]. The reads corresponding to choanoflagellates or Bicosoecophyceae were very few (relative abundance: 0% up to 0.016%) in these samples and were not analyzed further. The richness of haptophytes was comparable between the Osaka Bay and Uranouchi samples (Figure S5).

## 4. Discussion

The aim of the present study was to optimize experimental protocols for the MEGAPRIMER approach for ecological profiling of *Mimiviridae*. The new protocols were designed to require a small amount of DNA and streamline the experimental procedures. The initial work of MEGAPRIMER [23] and a follow up study [22] used individual primer pairs separately (i.e., MP1 method). Different primer cocktail strategies (MP1, MP5, MP10, and MP20) were applied to the Osaka Bay DNA sample that was also used in the original MEGAPRIMER report [23]. We demonstrated that all of the primer cocktail strategies reproduced similar *Mimiviridae* communities from this sample (Figure 2 and Figure 3, Figure S2). The analyses on three Uranouchi Inlet samples (UF, UJ, and UM) revealed distinct OTU profiles (Figure 2). The UJ sample was analyzed by both MP1 and MP10 methods, which again yielded similar OTU profiles (Figure 3).

In our assessment, MP10 showed the highest performance in terms of the numbers of *Mimiviridae* reads and the taxonomic richness (Figure 4, Table 2). In contrast, MP1 showed the highest read usability, but it required the largest amount of DNA (i.e., 82 ng). MP20 had an obvious advantage in requiring the smallest amount of DNA (i.e., 3.13 ng), but showed the lowest proportion of MAPS-validated *Mimiviridae* reads. Considering all aspects of the experimental and analytical performance for the Osaka Bay sample, we chose MP10 as the best choice for further experiments on the Uranouchi Inlet samples. We also identified the purification step can be critical for the outcome of sequencing. Purification with ethanol precipitation combined with gel extraction (protocol 3) was effective for all tested samples, though the S-OB-MP10-1 dataset exhibited a relatively low (but acceptable) proportion of MAPS-validated reads (Table 2). In contrast, purification with magnetic beads (protocol 1, 2, 4, 5) worked for the Osaka Bay sample but not for the Uranouchi Inlet samples. The protocol 3 is thus suggested as the best choice based on the presented results. MP10.v2 facilitated quality control during our protocol development. However, we did not design our experiment to compare the effectiveness between MP10.v1 and MP10.v2; we did not perform MP10.v1 combined with the protocol 3 for the Uranouchi Inlet samples. To summarize, primer cocktail methods reproduced the MP1 methods, with the advantage that they were much less laborious and required smaller amounts of DNA.

The D-OB-MP1-0 dataset was previously determined and is composed of a much larger number of *Mimiviridae* reads and OTUs than other datasets (Table 2). Nonetheless, it showed a similar OTU composition with other Osaka Bay datasets (Figure 3). The slight difference between D-OB-MP1-0 and the other Osaka Bay datasets may be due to the difference in sequencing depths as well as the choice of primers. In the original report [23], 24 of the 82 primer pairs did not yield a visible band in an agarose gel and were not subjected to the subsequent sequencing. The D-OB-MP1-2 dataset generated by the present study contained reads corresponding to nine of these 24 primer pairs (Figure S6). In contrast, the D-OB-MP1-0 dataset showed reads corresponding to six primer pairs that were missed in the D-OB-MP1-2 dataset.

We designed qPCR primers targeting six OTUs discovered by MEGAPRIMER barcoding and applied the primers to quantify the abundances of these OTUs in each sample. We successfully confirmed the presence of the OTUs in our samples. The sum of the measured *polB* gene concentrations ranged between 0.7 × 10^3^ and 2.6 × 10^3^ molecules mL^–1^ in the four samples. These values are in good agreement with the previous estimation of the concentration of NCLDV genomes in oceanic waters [16]. In their study, Hingamp and colleagues used bacterial cell counts based on flow cytometry and microscopic analyses, and NCLDV genome concentrations were estimated based on the metagenome derived ratio between NCLDV to bacterial marker genes. They also estimated that approximately 36% of the NCLDV genomes in the analyzed samples were *Mimiviridae*. This figure lead to an estimation of the concentration of *Mimiviridae* genomes between 1.4×10^3^ and 6.1×10^4^ genomes mL^–1^ (1.6 × 10^4^ genomes mL^–1^ on average) for the bacterial size fractions for photic zone samples.

As expected from a previous study [23], we observed the possible PCR amplification bias by MEGAPRIMER. Indeed, relative frequencies of several OTUs in a specific sample assessed by the MEGAPRIMER analysis were incongruent with the qPCR quantification of the OTUs. This discrepancy may originate from the high degeneracy of the primers. Li et al. showed that some OTUs can be amplified by up to 38 primer pairs, while other OTUs may only be amplified by a single primer pair [23]. We also suspected that the clustering of OTUs at 97% nucleotide sequence identify contributed to this discrepancy, because OTUs could include multiple genotypes which may differ at the qPCR primer regions. We therefore repeated our analysis using the OTU cut-off of 100% nucleotide identity and compared it with the qPCR results. However, we found no notable differences from the results obtained by the 97% OTU level analysis (Figure S7).

Despite these possible amplification biases among different OTUs, the relative frequency profiles for individual OTUs across multiple samples were reminiscent of those derived by qPCR quantification (Figure 5). For OTU1610 and OTU231, which were quantified in two or more samples by qPCR, the read frequency profiles across different samples were similar to those obtained by qPCR (Figure 5). These results imply that the read frequencies of OTUs revealed by the MEGAPRIMER approach still contain information about the true relative abundances of the OTUs, especially when they were compared between samples (not between OTUs in a single sample). We anticipate that the limit and effectiveness of the MEGAPRIMER approach will be further clarified through comparisons of larger datasets.

We found that *Mimiviridae* richness and community compositions were different between Osaka Bay and Uranouchi Inlet samples (Figure 1, Figure 2 and Figure 3). The Osaka Bay sample was on average twice as rich as Uranouchi Inlet samples. Previous studies also showed that samples from Osaka Bay displayed a higher *Mimiviridae* richness compared with other environmental samples (i.e., seawater, brackish water, and hot spring water) [22,23]. As viruses are dependent on their hosts for their reproduction, we hypothesized that the high *Mimiviridae* richness in the Osaka Bay sample may reflect a high host richness/diversity in the sample. Indeed, the Osaka Bay sample showed a larger Shannon’s diversity index (4.1) than the three Uranouchi Inlet samples (2.6–3.3). However, other characteristics did not reveal any convincing evidence for the high richness of *Mimiviridae* in the Osaka Bay sample. Regarding eukaryotic OTU richness, the Osaka Bay sample was richer than two Uranouchi samples (UF and UJ) but less rich than one Uranouchi sample (UM). Of the six major eukaryotic lineages shown in Figure 6C, only Chlorophytes are known to be hosts for *Mimiviridae*, although the hosts of the highly diverse environmental *Mimiviridae* are mostly unknown. It is interesting to note that the Osaka Bay sample showed the highest proportion of Chlorophytes among the compared samples. However, we did not find any particular increase of richness in the major eukaryotic lineages or other lineages corresponding to potential hosts of *Mimiviridae* (Figure S5). Therefore, it was difficult to conclude the source of the high *Mimiviridae* diversity in Osaka Bay in the present study.

In conclusion, we tested primer cocktail methods for the MEGAPRIMER approach and showed that primer cocktails reproduced the results obtained by the MP1 approach. The primer cocktail methods (i.e., MP5, MP10, MP20) reduced the required amount of environmental DNA and sample preparation time. These newly developed methods will facilitate the use of the MEGAPRIMER approach and will help to scale up studies of ecological characterization of *Mimiviridae*.

## Figures and Tables

**Table 1:** Datasets produced in this study.

Dataset	Sequencing Run Number	Sampling Location	Sampling Date	Primer Cocktail	Protocol Number^1^
D-OB-MP5-1	1	OB	2015.10.30	MP5	1
D-OB-MP10-1	2	OB	2015.10.30	MP10.v1	1
D-OB-MP20-1	3	OB	2015.10.30	MP20	1
D-OB-MP1-2	4	OB	2015.10.30	MP1 (no mix)	2
D-OB-MP5-2	5	OB	2015.10.30	MP5	1
D-OB-MP10-2	6	OB	2015.10.30	MP10.v1	1
D-OB-MP20-2	7	OB	2015.10.30	MP20	1
S-OB-MP10-1	8	OB	2015.10.30	MP10.v2	3
S-OB-MP10-2	8	OB	2015.10.30	MP10.v1	1
S-OB-MP10-3	8	OB	2015.10.30	MP10.v2	4
S-OB-MP10-4	8	OB	2015.10.30	MP10.v1	5
S-UF-MP10	8	UF	2017.6.21	MP10.v2	3
S-UJ-MP1	8	UJ	2017.7.6	MP1 (no mix)	3
S-UJ-MP10	8	UJ	2017.7.6	MP10.v2	3
S-UM-MP10	8	UM	2017.11.10	MP10.v2	3

^1^ Samples were processed with five different protocols. The difference among protocols are detailed in Supplementary Table S6.

**Table 2:** Overview of the generated *Mimiviridae polB* amplicon data.

Dataset	Number of Raw Reads	*Mimiviridae* Reads	Proportion of *Mimiviridae* Reads	Number of OTUs	Primercocktail	Protocol Number
D-OB-MP1-0 [23]	16,677,495	8,432,837	51%	5,595	MP1 (58/82 primer pairs)	-
D-OB-MP5-1	5,078,212	992,088	20%	3,018	MP5	1
D-OB-MP10-1	5,995,548	1,916,193	32%	3,396	MP10.v1	1
D-OB-MP20-1	10,720,091	1,019,645	10%	3,110	MP20	1
D-OB-MP1-2	2,205,016	497,356	23%	2,608	MP1	2
D-OB-MP5-2	2,992,984	273,153	9%	2,426	MP5	1
D-OB-MP10-2	4,521,841	340,129	8%	2,912	MP10.v1	1
D-OB-MP20-2	4,752,035	452,365	10%	2,755	MP20	1
S-OB-MP10-1	60,348	5,258	9%	744	MP10.v2	3
S-OB-MP10-2	78,067	37,638	48%	1,487	MP10.v1	1
S-OB-MP10-3	34,860	11,942	34%	1,243	MP10.v2	4
S-OB-MP10-4	38,477	21,965	57%	1,388	MP10.v1	5
S-UF-MP10	96,149	29,275	30%	601	MP10.v2	3
S-UJ-MP1	67,990	19,151	28%	470	MP1	3
S-UJ-MP10	68,168	31,276	46%	539	MP10.v2	3
S-UM-MP10	82,516	18,911	23%	595	MP10.v2	3

**Table 3:** Overview of 18S rRNA gene data.

Sampling Location	Number of Raw Reads	Taxonomically Annotated Reads	Number of OTUs
OB	67,028	44,727	439
UF	95,352	63,833	325
UJ	81,281	67,479	285
UM	80,237	54,845	528
Total	323,898	230,884	1,156

## Supplementary Materials

The following are available online at https://www.mdpi.com/2076-2607/8/4/506/s1, Figure S1: The richness of the *Mimiviridae polB* OTUs from all datasets, Figure S2: Phylogeny of *Mimiviridae* polB OTUs and relative frequency profiles in datasets, Figure S3: Occurrence of OTUs across samples, Figure S4: Relative read frequencies and concentrations of six OTUs selected for qPCR assessment, Figure S5: OTU richness of major eukaryotic lineages in four samples, Figure S6: Comparison of MP1 results between the previous and present studies, Figure S7: Relative read frequencies and qPCR quantifications for 100% nucleotide identity OTUs, Table S1: MEGAPRIMER DNA sequences, Table S2: Mixing scheme for MP5, Table S3: Mixing scheme for MP10 version 1, Table S4: Mixing scheme for MP10 version 2, Table S5: Mixing scheme for MP20, Table S6: *Mimiviridae polB* amplification protocols, Table S7: *Mimiviridae* OTUs that were quantified with qPCR, Table S8: Major lineages of the 18S rRNA gene analysis.
