# Supplementary material for: An Optimized Metabarcoding Method for Mimiviridae
Source: Microorganisms. 2020 Apr 2;8(4):506. doi: 10.3390/microorganisms8040506 (PMC7254495; doi:10.3390/microorganisms8040506)
Supplement: Supplementary file 1 [file microorganisms-08-00506-s001.zip › supplementary files/supplementary_figures-738930.pdf]

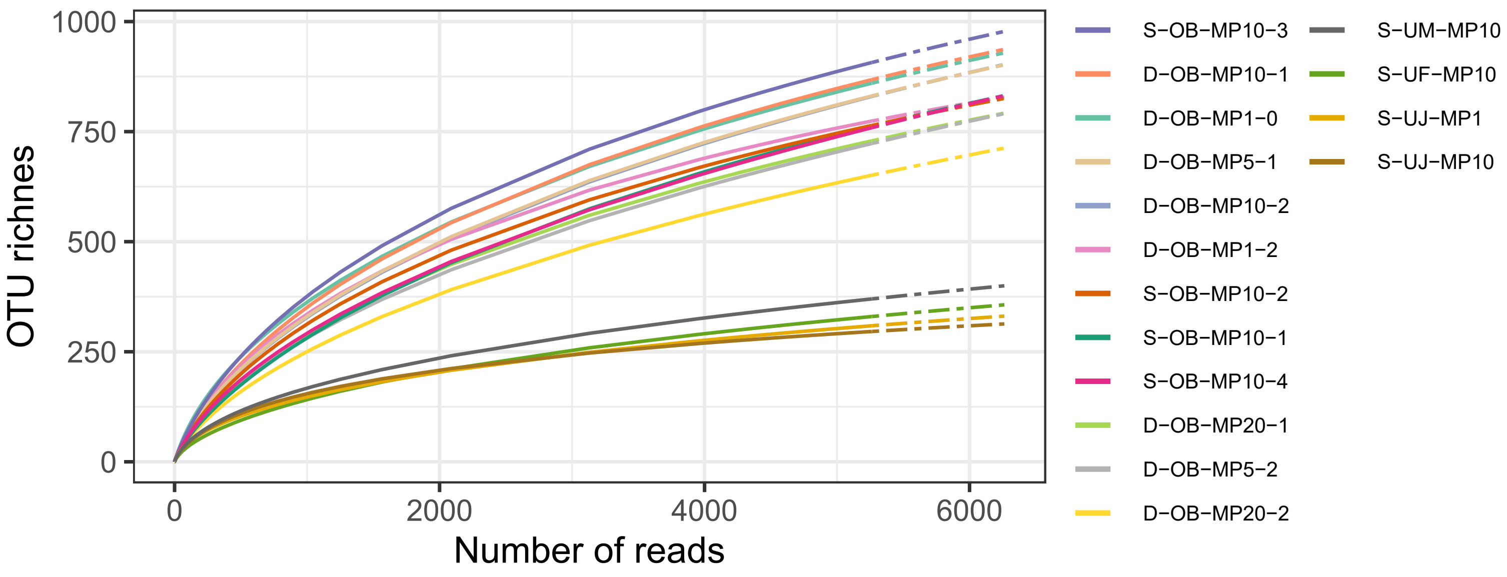

Figure S1. The richness of the *Mimiviridae polB* OTUs from all datasets. Datasets were subsampled for the purpose of sequence-depth normalization.

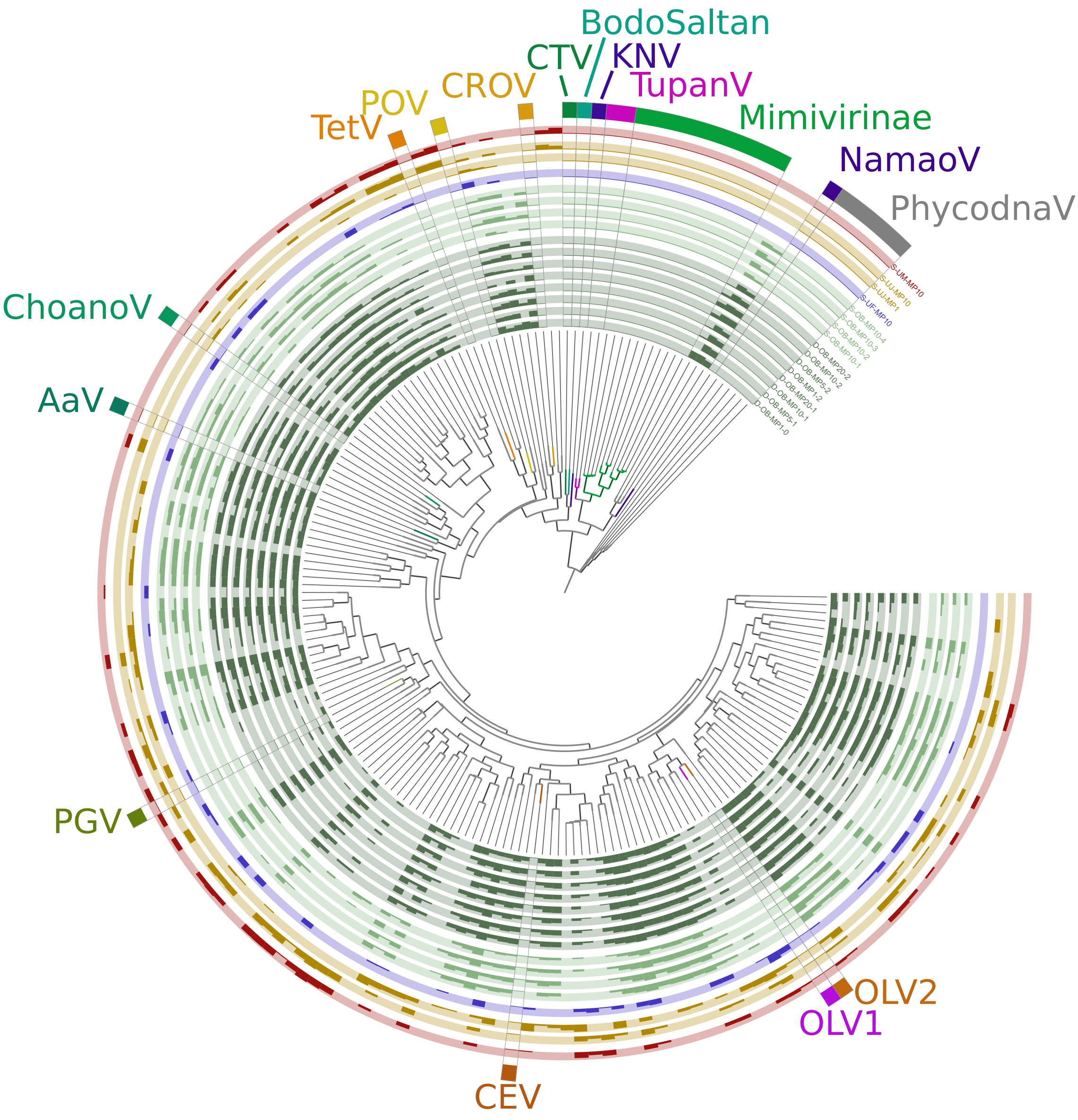

Figure S2. Phylogeny of *Mimiviridae* polB OTUs and relative frequency profiles in datasets. The maximum-likelihood phylogenetic tree based on amino acid sequences shows phylogenetic relationships among the most abundant 25 OTUs from each of the 16 datasets. The tree shows 150 OTUs with 26 *Mimiviridae* reference sequences and six *Phycodnaviridae* sequences as outgroup. Branches for reference sequences are highlighted with colors. The tree is unscaled. Color-coded rings outside the tree represent relative frequencies (in logarithmic scale) of the OTUs in 16 datasets. Dark green, Osaka Bay deep sequencing (including ds-OB-MP1-0 [23]); light green, Osaka Bay shallow sequencing; yellow, ss-UF-MP10; red, ss-UM-MP10; blue, ss-UJ-MP10/MP1. Reference sequences are as follows: CTV (Catovirus, KY684084), BodoSaltan (Bodo saltans virus, MF782455), KNV (Klosneuvirus, KY684109), TupanV (Tupanvirus deep ocean, MF405918.1; Tupanvirus soda lake, KY523104.1), Terravirus 2 TAO-TJA (GU265562), Mimivirinae (*Acanthamoeba polyphaga mimivirus*, NC_014649; Hirudovirus strain Sangsue, KF493731; Mimivirus pointerouge 1 strain Pr1, JF979167; Moumouvirus ochan, JQ063130; Moumouvirus monve isolate Mv13-mv, JN885998; Acanthamoeba polyphaga moumouvirus, NC_020104; Mimivirus Bus strain RTM2, JF979178; Megavirus courdo7 isolate Mv13-c7, JN885991; *Megavirus chiliensis*, NC_016072; Megavirus lba isolate LBA111, NC_020232), NamaoV (Namao virus, MG745875.1), PhycodnaV (Micromonas pusilla virus 12T, HQ632826.1; Micromonas sp. RCC1109 virus MpV1, NC_014767; Ostreococcus tauri virus 2, FN600414; Ostreococcus tauri virus 1, NC_013288; Ostreococcus lucimarinus virus 1, NC_014766; Bathycoccus sp. RCC1105 virus BpV2, HM004430), OLV2 (Organic lake phycodnavirus 2, HQ704803), OLV1 (Organic lake phycodnavirus 1, HQ704802), CEV (Chrysochromulina ericina virus isolate 01, EU006632), PGV (Phaeocystis globosa virus strain 16T, NC_021312), AaV (Aureococcus anophagefferens virus, YP_009052217.1), ChoanoV (Mimiviridae sp. ChoanoV1, MK250085.1), TetV (Tetraselmis virus 1, KY322437.1), POV (Pyramimonas orientalis virus isolate 01, EU006633), CROV (Cafeteria roenbergensis virus BV-PW1, NC_014637).

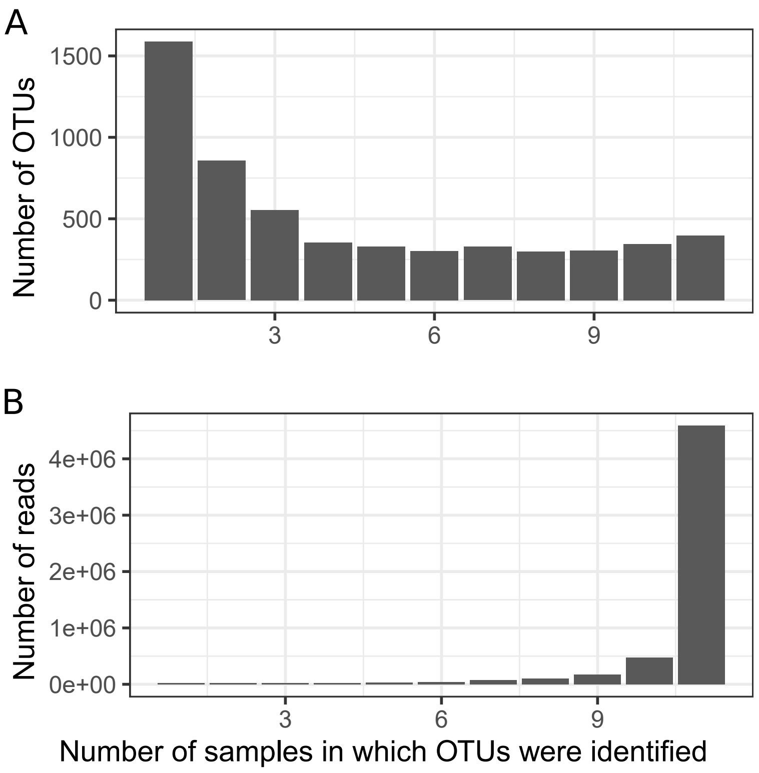

Figure S3. Occurrence of OTUs across samples. In these plots, OTUs are binned by the number of datasets (among the eleven Osaka Bay datasets) in which they were identified. The upper panel shows the number of OTUs for different bins. For example, the bar on the far left side indicates the number of OTUs that were identified in only one dataset, while the bar on the far right side indicates the number of OTUs found in all eleven datasets. The lower panel shows the number of reads in each bin.

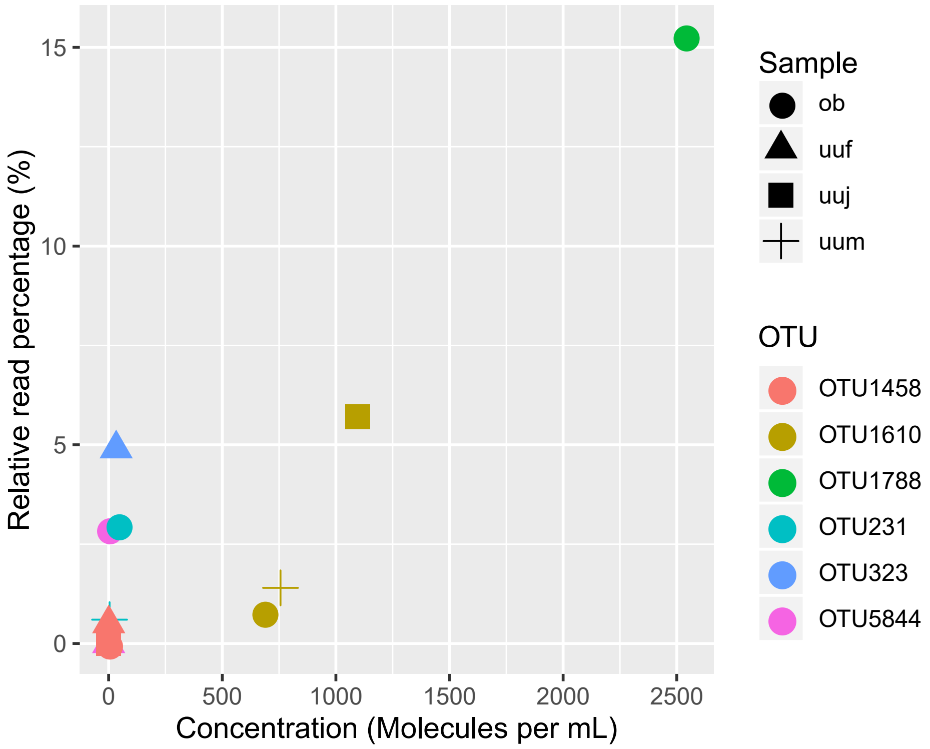

Figure S4. Relative read frequencies and concentrations of six OTUs selected for qPCR assessment. qPCR measurements that were below the LoD were omitted from the plot.

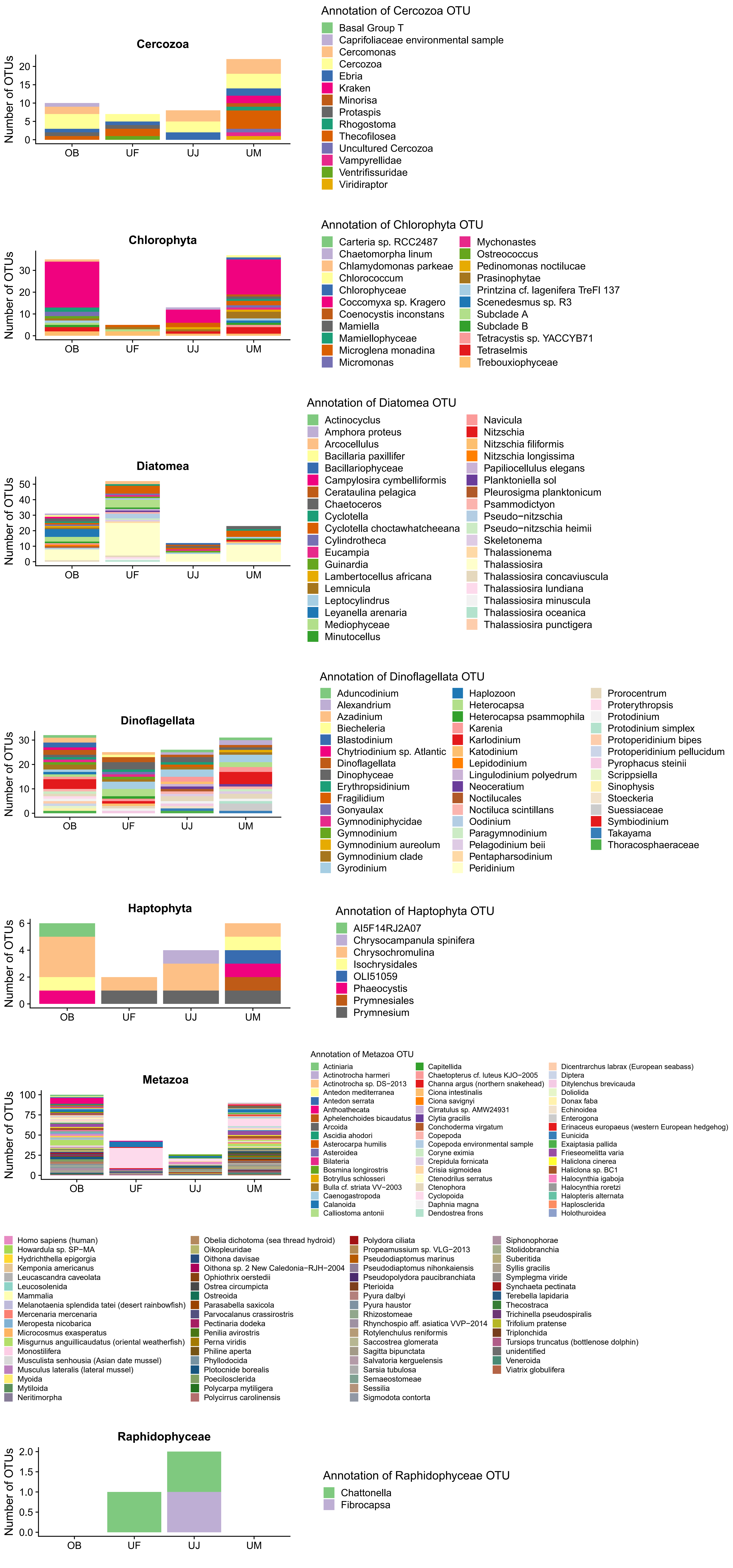

Figure S5. OTU richness of major eukaryotic lineages in four samples. The datasets were subsampled at 30,000 reads before the richness was calculated.

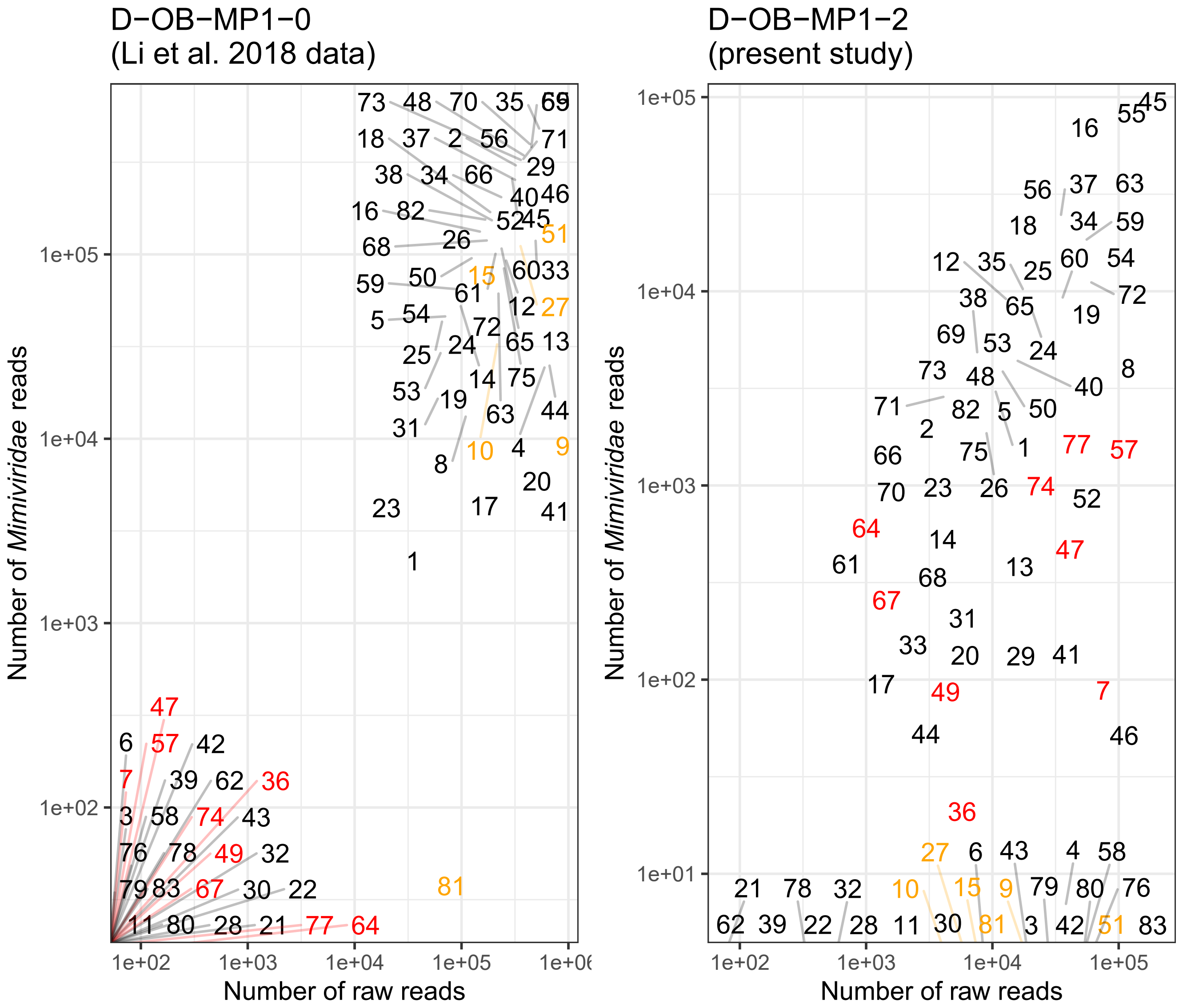

Figure S6. Comparison of MP1 results between the previous and present studies. In the previous study [23], amplicons based on 24 of the 82 primer pairs were visualized on agarose gel for the Osaka Bay sample, thus were not subjected to sequencing. However, of these 24 primer pairs, nine primer pairs (shown in red) did produce Mimiviridae reads in the present study. Six primers (shown in orange) did not produce reads in the MP1 experiment of this study (D−OB−MP1−2). Horizontal axes represent the number of raw reads, while vertical axes represent the number of reads annotated as *Mimivirdae polB*. Plots are in logarithmic scale with base 10.

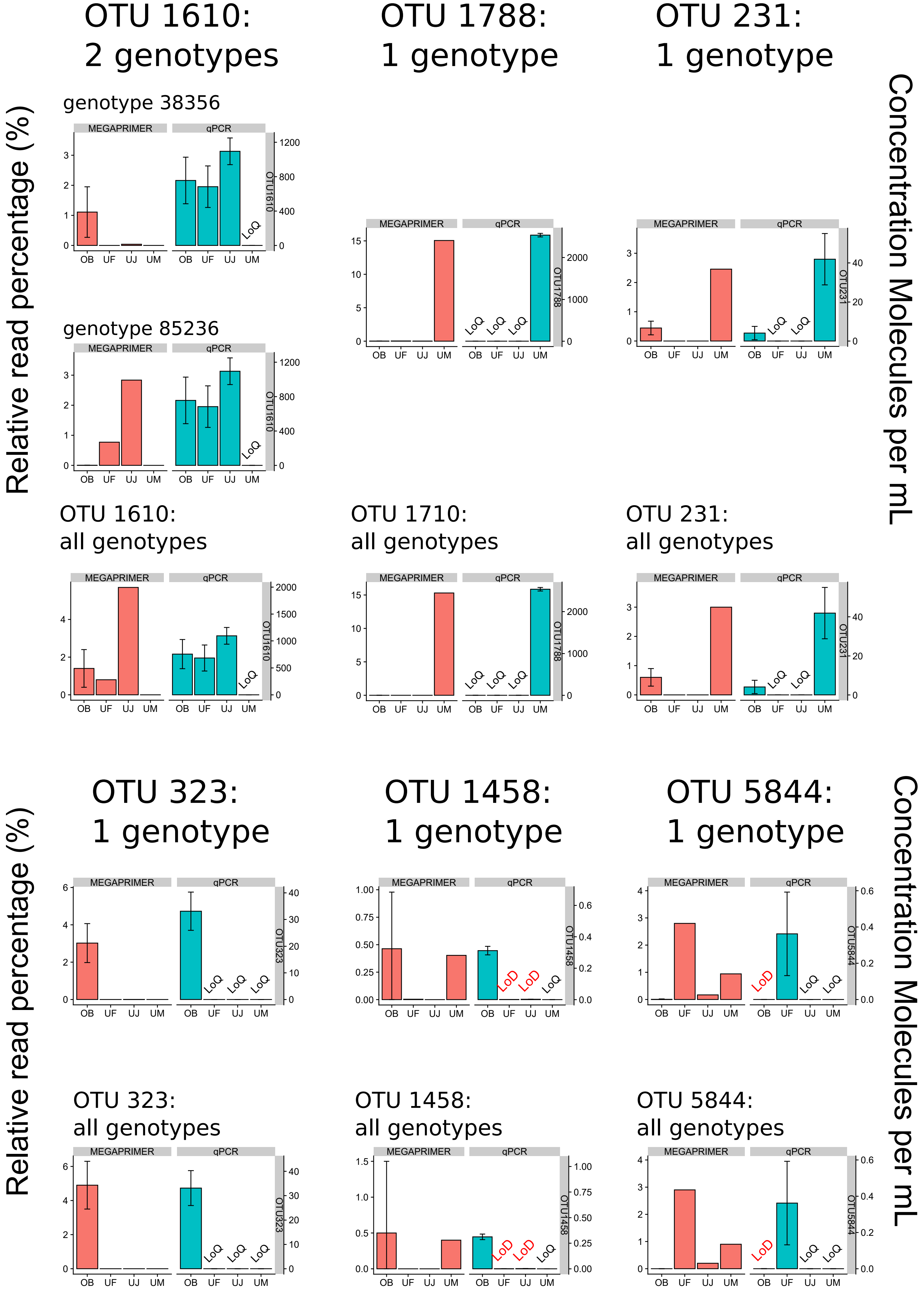

Figure S7. Relative read frequencies and qPCR quantifications for 100% nucleotide identity OTUs. Relative read frequencies and qPCR quantifications of the dominant genotypes and all genotypes for the selected six OTUs in four samples.
